# Biological Mechanisms Linking Social Adversity and Cognition

**DOI:** 10.3389/phrs.2025.1608740

**Published:** 2025-11-05

**Authors:** Aileen Liang, Emma Watt, Noha Gomaa

**Affiliations:** Schulich School of Medicine and Dentistry, Western University, London, ON, Canada

**Keywords:** social adversity, cognitive health, inflammation, allostatic load, epigenetics

## Abstract

**Objectives:**

Various studies have shown that social adversity, such as loneliness or low SES, are linked with worse cognitive outcomes, though underlying biological mechanisms remain unclear. This scoping review aims to summarize existing evidence on biological processes that may serve as mediators underlying this association.

**Methods:**

Following PRISMA-ScR guidelines, studies measuring social adversity, cognition, and at least one biological mechanism were included. Results were summarized narratively and in tabular formats.

**Results:**

Twelve studies (n = 12) examined links between social adversity, cognition, and biological mechanisms. Inflammation, allostatic load, genetics and genetic aging markers were the three main biological mechanisms identified as potential mediators.

**Conclusion:**

Several studies suggest that these biological mechanisms may mediate the link between social adversity and cognitive decline. However, further research is needed to clarify these complex relationships, which are crucial for developing targeted interventions, especially for socially disadvantaged populations.

## Introduction

Aging is a multifaceted process that encompasses physical, cognitive, and emotional changes. As the global population continues to age, understanding the factors that influence cognitive health in later life has become a critical area of research. Increasing evidence highlights the significant role of social exposures, such as social engagement, socioeconomic status (SES), social support, and social networks, in shaping cognitive trajectories during aging [1, 2]. For an example, studies have consistently shown that social engagement is protective against cognitive decline, with older adults who maintain active social lives exhibiting better cognitive performance and a reduced risk of dementia [1, 3]. Conversely, social isolation and loneliness have been linked to an increased risk of cognitive impairment and dementia [3, 4]. In a longitudinal study, Wilson and colleagues found that individuals with higher levels of social activity were less likely to develop Alzheimer’s disease, suggesting that cognitive stimulation from social interactions may help preserve brain function in aging [5]. The association between social networks and cognitive health is further supported by studies showing individuals with broader and more supportive social ties to experience slower rates of cognitive decline [2, 6]. In the coming decades, older adults are expected to make up a third of Canada’s population, and cognitive impairments can have profound negative impacts on an older adult’s life [7]. One cohort study showed associations between mid-life marital status and later cognitive functions, where being divorced or widowed were both associated with greater odds of later cognitive impairments [8]. Another cohort study showed social isolation and loneliness in older adults are both associated with lower cognitive scores [9]. SES also plays a critical role in cognitive health during aging. Low SES, characterized by limited access to resources, education, and healthcare, has been consistently associated with worse cognitive outcomes and a higher risk of dementia [3]. The impact of SES on cognitive aging is postulated to be mediated through various pathways, including chronic stress, limited access to healthcare, and less cognitive stimulation throughout life [10]. These findings underscore the importance of social environments and experiences in shaping cognitive trajectories during aging, pointing to the need for integrated approaches that consider both individual and social factors. However, despite the wealth of evidence supporting the link between social exposures and cognitive outcomes in older adults, the mechanisms that underlie these associations remain unclear.

The biopsychosocial model of health, which was first proposed by Engel in 1977, emphasizes the interaction between biological, psychological, and social factors in influencing health outcomes [11, 12]. In the context of cognitive aging, this model provides a comprehensive framework for understanding how social exposures contribute to cognitive function. Social factors, such as social support and engagement, interact with psychological and biological processes to influence cognitive outcomes. For instance, psychological factors like stress, coping strategies, and mental health are known to interact with social support to affect cognitive health [1, 13]. Chronic stress, often exacerbated by adverse social environments, has been implicated in cognitive decline through its effects on the hypothalamic-pituitary-adrenal (HPA) axis, leading to elevated levels of cortisol that can negatively impact brain regions such as the hippocampus, which is crucial for memory and learning [14]. Moreover, social engagement may buffer against the negative effects of stress by promoting positive psychological states, such as a sense of belonging and self-worth, which, in turn, may protect against cognitive decline [15, 16]. At the biological level, several mechanisms have been proposed to explain how social exposures may influence cognitive aging. One of the most studied mechanisms is the role of inflammation. Chronic stress and social adversity have been shown to increase inflammatory markers, which in turn may contribute to neurodegenerative processes and cognitive decline [17, 18]. Inflammatory responses, particularly the activation of microglia and the release of proinflammatory cytokines, have been linked to brain aging and Alzheimer’s disease pathology [19]. Another potential biological pathway is neurotrophic support. Social engagement has been shown to enhance the release of brain-derived neurotrophic factor (BDNF), a protein crucial for neuronal survival and plasticity, thereby promoting cognitive resilience in the face of aging [20]. Another potential mechanism linking social exposures to cognitive health in older age is through epigenetics [21, 22]. Studies over the last decade have shown that age-related epigenetic changes quantified in epigenome-wide association studies or those characterizing the epigenetic clock are linked to cognitive health in older age and may be modified by social exposures. Other mechanisms that have been explored include increased allostatic load, which is defined as the “wear and tear” the body experiences when repeated neural or neuroendocrine responses are activated during stressful situations, as well as telomere shortening [14, 23–25].

This scoping review aims to summarize the existing evidence on the association between measures of social adversity and neurocognitive outcomes in adults and describe potential the underlying biological processes and pathways. Specifically, we will summarize evidence on diverse but interconnected biological mechanisms including inflammation, allostatic load, and genetic and epigenetic markers as potential mediators for the association often seen between social adversity and cognition. By mapping the existing evidence and identifying key research areas, we hope to contribute to a deeper understanding of how social, psychological, and biological factors converge to influence cognitive outcomes in later life. This knowledge is crucial not only for advancing the science of cognitive aging but also for developing targeted interventions that address the social determinants of cognitive health and that promote healthy aging.

## Methods

This manuscript was developed in accordance with the Preferred Reporting Items for Systematic Reviews and Meta-Analyses extension for Scoping Reviews (PRISMA-ScR) Checklist [26]. A review protocol can be accessed on OSF registries (DOI 10.17605/OSF.IO/2DFWM).

### Eligibility Criteria

Eligible studies examined human adult populations and included measures of social adversity as exposures, measures of cognitive outcomes, and measures of biological mechanisms that link exposure and outcome. Social adversity is defined according to the WHO Commission on the Social Determinants of Health Framework which includes income and social protection, education, unemployment and job insecurity, working life conditions, food insecurity, housing, basic amenities, and environment, social inclusions and non-discrimination [27]. We did not include early childhood development. Cognitive measures included any studies who assessed either general cognition or a specific cognitive domain [3, 28]. All study designs were included. Animal studies, and those that focused on pediatric populations, and studies not in the English language were excluded. We also excluded the grey literature, editorials, and other reviews or narrative articles.

### Data Sources and Search Strategy

A research librarian at Western University libraries was consulted during the development of the search strategy. Relevant literature identified through systematic searches conducted in databases MEDLINE, Embase, PsychINFO (all via Ovid interface), and Scopus. Keywords and medical subject headings (MeSH) were used to identify relevant studies, and the cutoff date for the search was March 5, 2024. Reference lists of included studies were also manually screened to ensure a comprehensive search. The complete search strategy is available in the Supplementary Appendix S1.

### Study Selection and Screening

All literature search results were uploaded to Covidence software and duplicates were removed through both manual screening and an automated duplication check conducted by Covidence. The two reviewers (AL and EW) then independently conducted a title abstract screening of the eligible retrieved articles using Covidence. Studies deemed potentially relevant then underwent full text screening by both reviewers independently. After each level of screening, discrepancies were resolved during a consensus meeting between the two reviewers. Cohen’s kappa (K) coefficient was calculated for each level of screening to assess for inter-rater reliability in screening obtaining a coefficient range of 0.68–0.81 indicating substantial agreement between the reviewers.

### Data Charting and Synthesis

Data was extracted from the included studies independently by the two reviewers. Extracted data included study characteristics (authors, publication date, study design, location, number of participants), study variables (social adversity measure(s), cognition measure(s), biological mechanism measure(s)), and study outcomes (associations between studied variables).

### Evidence Appraisal and Data Synthesis

The Newcastle-Ottawa Scale was used to assess the risk of bias and quality (12). Completed risk-of-bias table is in the Supplementary Appendix S2. Study findings were summarized in a tabular format. A narrative summary of the results was also provided in line with the Synthesis Without Meta-analysis (SWiM) guidelines and following the PRISMA-ScR checklist (14).

## Results

### Study Selection and Characteristics

**FIGURE 1:**
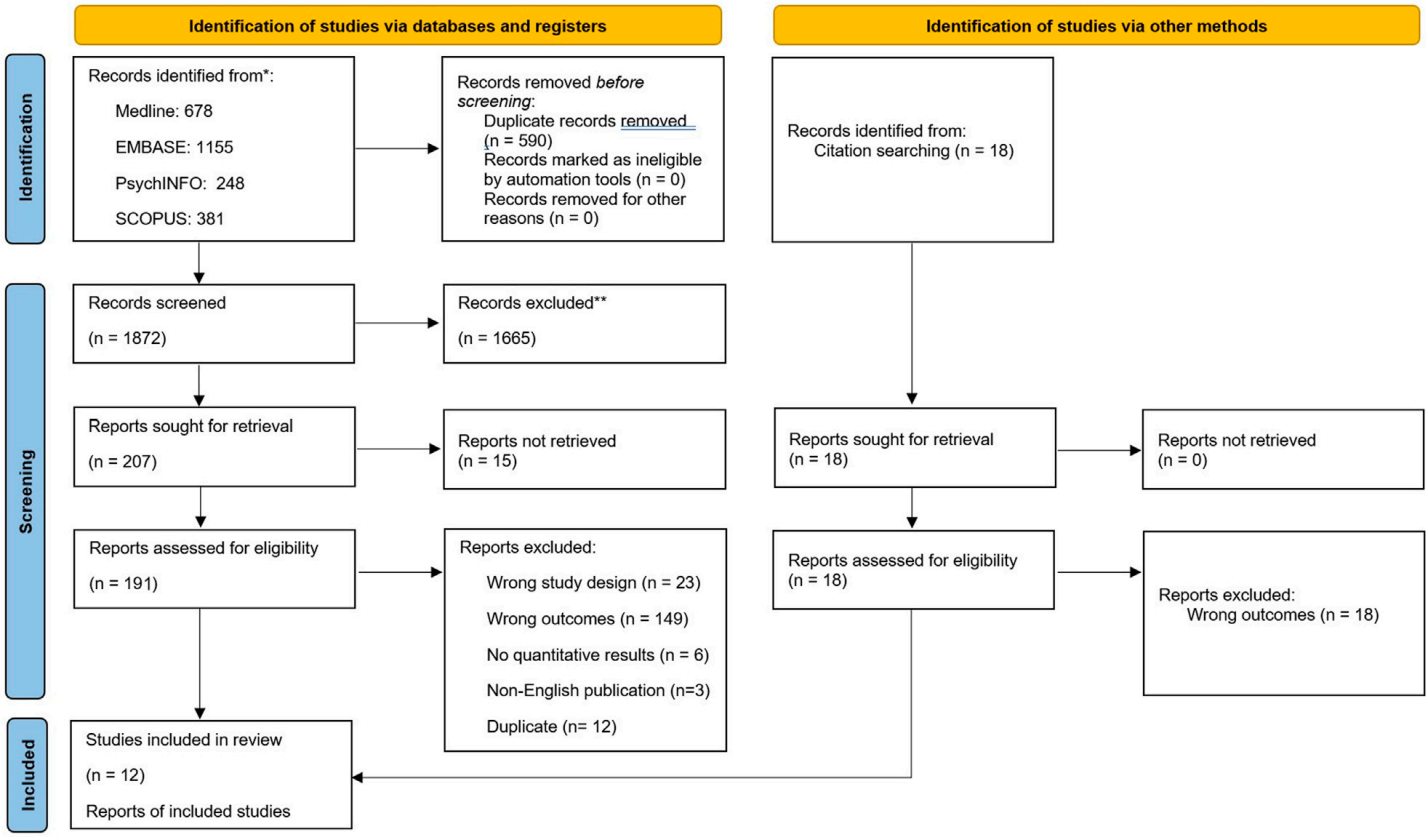
PRISMA flow diagram (Canada, 2025).

**FIGURE 2:**
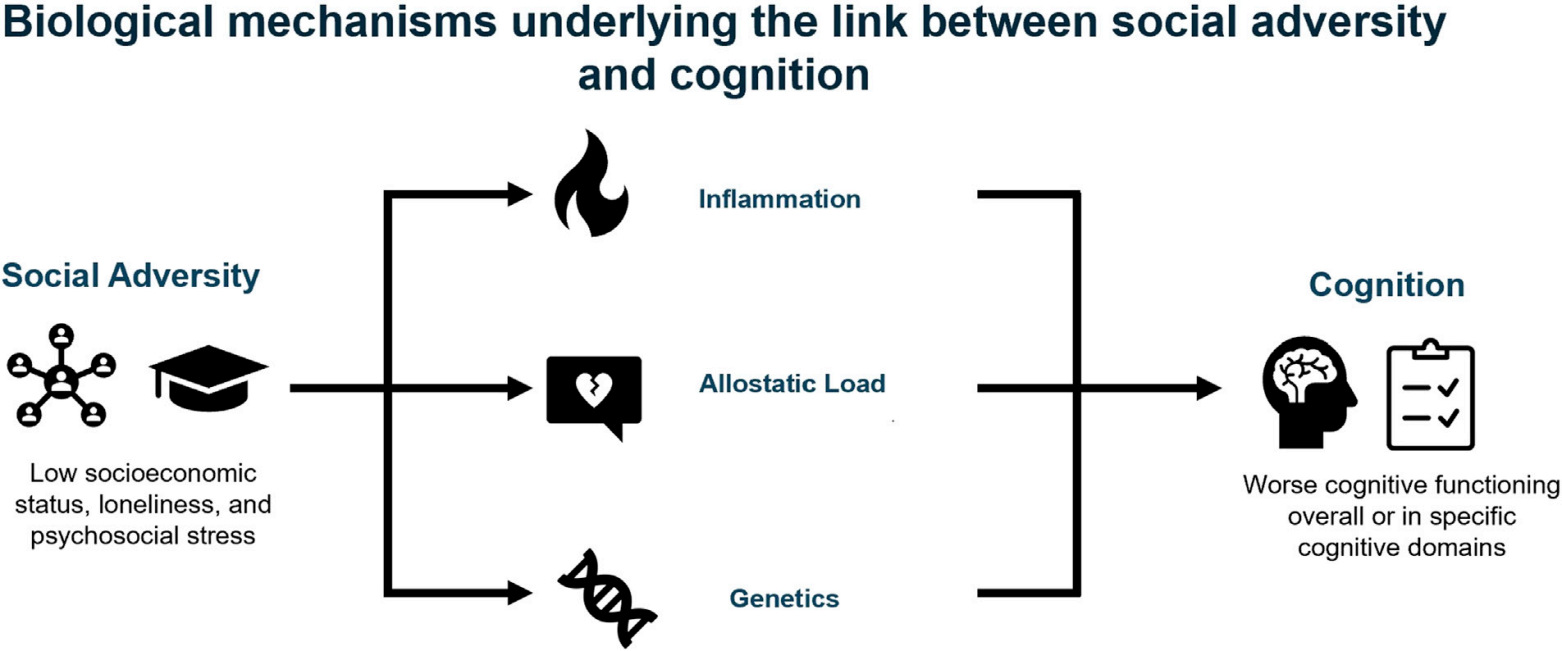
Visual abstract (Canada, 2025).

The search strategy yielded 1872 results. After title and abstract screening, 207 studies remained and underwent full-text screening, out of which 13 studies met the inclusion [16, 29–40]. Figure 1 summarizes the evidence of the studies screened and exclusions at each stage. The overall results of the study are summarized in the visual abstract (Figure 2).

The characteristics of included studies are outlined in Table 1. In assessing social adversity, six studies examined low SES, two examined loneliness, two focused on psychosocial stress, and other studies examined low social network, low social capital, social isolation, and social strain. To assess cognition, six studies looked at overall cognition measured by the Digital Symbol Substitution Test (DSST), Mini-Mental State Examination (MMSE), or telephone interview for cognitive status, for studies investigated specific domains of cognition including verbal fluency, attention, memory, motor skills, executive function, and processing speed. Two studies looked at cortical morphologies and white matter infrastructure using magnetic resonance imaging. Biological mechanisms included assessments of allostatic load, inflammation, and genetics or epigenetics or genetic aging markers. Overall findings from each of the studies included are summarized in Table 2. Below, we provide a narrative summarizing the biological mechanisms linking social adversity to cognitive outcomes based on the findings of the studies included in this review.

**TABLE 1:** Characteristics of included studies (Canada, 2025).

Study (author, year)	Journal	Study type	Location	n	Age	Study population	Social adversity measure	Description	Cognition measures	Description	Biological mechanism Measure	Description
Akrivos [29]	BMJ Open	cross-sectional	USA	3,234	>60	NHANES	Low SES	Education and poverty income ratio	Overall cognition	DSST	Allostatic load	SBP/DBP, BMI, WTR circumference, HDL, TC, HDL/TC, HbA1c, CRP
Boss [30]	Religions	cross-sectional	USA	88	>60	Housebound adults enrolled in MOW program	Stress and loneliness	Perceived Stress Scale and UCLA Loneliness Scale	Executive function	Clock draw	inflammation	Cortisol, CRP, Interleukin-1β
Fazeli [39]	Journal of Acquired Immune Deficiency Syndromes	cross-sectional	USA	96	>50	PLWH	Low SES	Education and income	Cognitive domains: Verbal fluency, attention, memory, motor skills, executive function	Controlled Oral Word Association Test, Animal Naming, Wisconsin Card Sorting Test, Trail Making Test A and B, Learning and Delayed Recall, Hopkins Verbal Learning Test, Brief Visuospatial Memory Test, DSST, Symbol Match, Letter Number Sequencing, Paced Auditory Serial Addition Test, Grooved Pegboard Test	Allostatic load	SBP/BMP, cortisol, DHEA, IL-6, TNF-alpha, C-reactive protein, glucose, total cholesterol, HDL cholesterol, triglycerides, albumin
Foverskov [40]	European Journal of Ageing	cross-sectional	Denmark	5,575	49–63	CAMB	Economic hardship	Household disposable income	Overall cognition	sentence completion, verbal analogies, number series	Inflammation	CRP, IL-6, TNF-α
Huang [31]	Psychoneuroendocrinology	cross-sectional	Singapore	353	65–80	SLAS-2	Low SES	Education and housing type	Overall cognition	MMSE	Genetics/epigenetics/genetic aging markers	Leukocyte telomere length (LTL)
Krishnadas [32],	Psychosomatic Medicine	cross-sectional	Scotland	42	35–64	Subjects selected based on SIMD	Socioeconomic deprivation		Cortical morphologies	cortical volume, thickness, surface area	Allostatic load	CRP, IL-6, triglycerides, HDL, VLDL, fibrionogen, D-dimer, insulin, BMI
Molesworth [33]	SCAN	cross-sectional	USA	155	30–50	community-dwelling adults in Pennsylvania	Low social network diversity and size	Participation in social roles and number of regular contacts	White matter infrastructure	White matter integrity and fractional anisotropy	inflammation	IL-6, CRP
Lynch [38]	J Gerontol B P	cross-sectional	USA	1814	50–98	HRS	Loneliness	UCLA Loneliness Scale	Overall cognition	Telephone Interview for Cognitive Status	Genetics/epigenetics/genetic aging markers	DNA methylation age acceleration, “GrimAge”
Liang [16]	Int. J. Aging Hum. Dev	cross-sectional	Canada	1,479	45–85	CLSA	Low social capital	Participation in community-related activities and MOS-Social Support Survey	Cognitive domains (attention, verbal fluency, memory, executive function, psychomotor speed)	Stroop Test, Animal Naming, Controlled Oral Word Association Test, Prospective Memory Test, Rey Auditory Verbal Learning Test, Mental Alternation Test, Simple and Choice Reaction Times	Genetics/epigenetics/genetic aging markers	DNA methylation age acceleration
De Looze [37]	Brain Behav Immun	longitudinal	Ireland	3,457	>50	TILSA	Perceived stress	Perceived Stress Scale	Cognitive domains (verbal fluency, memory)	Animal Naming, Immediate- and Delayed-Recall	allostatic load and genetics/epigenetics/genetic aging markers	SBP, DBP, RHR, PWV, waist-height ratio (WHR), body mass index (BMI), glycosylated haemoglobin (HbA1c), total cholesterol (TChol), high-density lipoprotein, CRP, Creatinine, Cyscatin C Leukocyte telomere length
Qi [35]	PNEC	cross-sectional	USA	2,535	>60	NHANES	Social isolation	Social Network Index	Overall cognition	DSST	inflammation	CRP, plasma fibrinogen, and serum albumin
Malatyali [36]	Gerontol. Geriatr. Med	cross-sectional	USA	9,262	>50	HRS	Social strain	Perceived Social Strain Scale	Overall cognition	Telephone Interview for Cognitive Status	inflammation	CRP

NHANES , national health and nutrition examination survey; IGEMS, the interplay of genes and environment across multiple studies; HRS, health and retirement study at university of michigan; PLWH, people living with HIV, CLSA, canadian longitudinal study on aging; MOW, meals on wheels; TILSA, the irish longitudinal study on ageing; SLAS-2, Singapore Longitudinal Ageing Study II, CAMB, copenhagen ageing and midlife biobank; SIMD, Scottish Index of Multiple Deprivation; DSST, digit symbol substitution test.

**TABLE 2:** Findings from included studies (Canada, 2025).

Study (author, year)	Association between cognition and social adversity	Association between biological mechanism and social adversity	Association between biological mechanism and cognition	Biological mechanism as mediator for association between social factor and cognition
Akrivos [29]	level of education and PIR was significantly positively associated with performance on the DSST	SES was significantly negatively associated with AL	AL was significantly negatively associated with cognitive performance	No mediation: AL was not a significant mediator of the SES-related differences in cognition, it mediated at most 4.5% of the SES effect on DDST performance in the highest PIR quartile
Boss [30]		Greater loneliness predicted greater CRP and had a near significant contribution to cortisol	IL-1β showed a significant positive correlation with executive function	
Fazeli [39]	SES was a significant predictor of cognitive functions	No significant associations emerged between SES and AL.	No significant associations between AL and cognition in the overall sample, but higher AL was associated with lower cognitive outcomes in African American PLWH.	Mediation present: The indirect effect of SES on neurocognitive functioning through allostatic load was significant, suggesting mediation
Foverskov [40]	Experiencing EH for more than 4 years was associated with significantly lower cognitive test scores	Four or more years in EH was related to higher inflammatory levels for CRP and Interleukin-6		
Huang [31]	High SES appears to protect individuals from age-related declines in cognitive function. There is significant negative association between age and cognitive function in lower SES levels but not higher SES levels	Among older adults with lower and mean SES levels, there is a significant age-related decline in LTL. In contrast, there is no significant association between age and LTL among individuals with higher SES.		Partial mediation: Specifically, older adults with lower SES experience large age-related decreases in cognitive functioning, which are then associated with shorter LTL. In contrast, participants with higher SES maintain higher cognitive functioning despite increasing age, which in turn is related to greater LTL.
Krishnadas [32]	The MD (most deprived) group had statistically significantly smaller volumes pertaining to the left posterior parietal cortex and right Broca homologue compared with the LD (least deprived) group			Mediation present: Inflammation factor mediated the relationship between deprivation status and left Wernicke’s region CT.
Molesworth [33]	There is a positive association between a measure of white matter integrity, fractional anisotropy (FA), and social network diversity, particularly near anterior corpus callosum	IL-6 was negatively associated with the diversity of a person’s social network	FA is weakly associated with levels of IL-6	No mediation: IL-6 did not mediate the social network and FA relationship
Lynch [38],	Significant effect of loneliness on overall cognition		Significant correlations between DNAm AgeAccel measures and cognition	Mediation present: GrimAge Accel consistently explained the association between loneliness and general cognitive ability, immediate recall, and delayed memory recall
Liang [16],	Lower structural social capital was significantly associated with worse attention and verbal fluency	Lower structural social capital was significantly associated with greater epigenetic age acceleration difference		
De Looze [37]	Higher perceived stress was significantly associated with worse cognitive outcomes		Longitudinally, AL was associated with lower cognitive functions	No mediation: The strength of associations between perceived stress and cognitive outcomes did not change when AL was adjusted
Qi [35]	Socially isolated older adults were found to have a poorer cognitive functioning than those not isolated	Social isolation was related to higher levels of CRP and fibrinogen in men		Mediation present: The association between social isolation and cognitive functioning mediated by CRP and fibrinogen was 6.1% and 12.0%, respectively
Malatyali [36]	Higher level of social strain from friends was significantly associated with the risk of CIND (cognitive impairment without dementia) and dementia		Higher levels of CRP were significantly associated with CIND, but not dementia	

### Inflammation

Five studies measured inflammation specifically and its relationship to social adversity and cognition. Qi et al. found that chronic inflammation was a mediator between social adversity and cognitive function. Specifically, they showed that socially isolated older adults have worse cognitive function measured by the DSST (β = −2.445, SE = 1.180, p < 0.01 for men; β = −5.478, SE = 1.167, p < 0.001 for women). For men specifically, the association between social isolation and cognitive functioning was mediated by c-reactive protein (CRP) and fibrinogen levels with the proportion mediated being 6.1% and 12.0%, respectively. On the other hand, Molesworth et al. assessed social networks and white matter microstructure, and found that, while there is an association between white matter integrity and social network diversity (mean = 0.012, p < 0.025), inflammatory cytokine interleukin-6 did not mediate this relationship (p > 0.1). Boss et al. found that both social adversity and cognition were independently related to inflammation. They found that greater loneliness correlated with CRP (r = 0.26, p = 0.02 and cortisol levels (p = 0.06), while executive function was correlated with interleukin-1β (r = 0.23, p = 0.03). Foverskov et al. similarly found that four or more years of economic hardship was related to higher levels of inflammatory biomarkers: 22% for CRP (95% CI [4, 44]) and 23% for interleukin-6 [95% CI 10., 39)]. Malatyali et al. found that CRP was related to mild cognitive impairment (β = 1.03, 95% CI [1.01, 1.06], p < 0.01) but not dementia [β = 1.021, 95% CI (0.98, 1.97)].

### Allostatic Load

Four studies examined various markers of allostatic load as it relates to social adversity and cognition. Fazeli et al. specifically examined older adults living with HIV and found SES was a significant predictor of neurocognitive functioning (β = 1.12, SE = 0.41, p = 0.008), and that allostatic load was a significant mediator of this relationship (β = 0.19, SE = 0.12, 95% CI [0.002, 0.485]). Similarly, Krishnadas et al. examined socioeconomic deprivation and measured cortical morphologies to find morphological differences between most-deprived and least-deprived groups, including a thinner left Wernicke’s area in the most-deprived (Cohen’s d = 0.93), and found inflammatory markers in particular mediated this correlation (fibrinogen, interleukin-6, CRP, and D-dimers). No other cardiometabolic factors mediated the relationship between deprivation status and Wernicke’s area cortical thickness [β = −0.029, SE = 0.15, 95% CI (−0.06 to −0.007)], which is the brain region responsible for language comprehension. Meanwhile, Akrivos et al. found that poverty and education were social factors that were significantly associated with outcomes on the Digit Symbol Substitution Test [β = 8.9, 95% CI (6.6 to 11.3), p < 0.0001], but that allostatic load was not a significant mediator of SES-related differences in cognition because it mediated at most 4.5% of the effects and only in the highest poverty income ratio quartile. Similarly, De Looze et al. found that perceived stress was significantly associated with cognition in the domain of verbal fluency and memory [β = −0.10, 95% CI (−0.12; −0.07), p < 0.001], but that the strength of these associations did not change when allostatic load was adjusted for (p = 0.13).

### Genetics, Epigenetics, and Genetic Aging Markers

Four studies examined different genetics and epigenetic markers as related to social adversity and cognition. Huang et al. and De Looze et al. both looked at leukocyte telomere length (LTL). Huang et al. found that for adults with lower SES levels, there was a negative association between age and cognitive function as measured by MMSE scores (β = 0.0339, SE = 0.0123, t = 2.75, p = 0.006). LTL served as a mediator specifically in adults with low SES, where age-related decreases in cognitive functions are associated with shorter LTL. On the other hand, De Looze et al., while also finding significant effect of perceived stress on cognitive function, did not find LTL to change the strength of these associations [*X* ^*2*^ (32) = 1.6, p =0.89]. Lynch et al. and Liang et al. both looked at DNA methylation age acceleration. Lynch et al. found that loneliness negatively impacts cognition. Specifically, GrimAge acceleration consistently mediated the relationship between loneliness and general cognitive ability, immediate recall, and delayed memory recall (β = −0.19). Similarly, Liang et al. found that epigenetic aging measured using Hannum clock in a large, population-based Canadian sample was independently associated with low structural cognitive capital [β = −0.79, 95% CI (−1.5, −0.19), p < 0.05].

## Discussion

This scoping review sought to investigate the biological mechanisms through which social adversity may impact cognitive outcomes during aging. Collectively, the findings indicate that various forms of social adversity—including low socioeconomic status (SES), loneliness, and psychosocial stress—are associated with negative cognitive outcomes, particularly in older adults. Importantly, biological mechanisms such as inflammation, allostatic load, and genetic/epigenetic markers were identified as potential mediators in these relationships, though the strength and consistency of these associations varied across studies. Inflammation appeared to play a prominent role, with several studies demonstrating that higher levels of inflammatory markers, such as CRP and interleukin-6, were linked to both social adversity and cognitive decline [30, 35]. However, other studies did not find inflammation to mediate the relationship between social adversity and cognition, highlighting the complexity of these interactions [33]. These mixed findings suggest that while inflammation is a plausible biological pathway, it may not be universally relevant for all individuals or social contexts, and further research is needed to better delineate its role in cognitive aging.

Allostatic load, a measure of the cumulative physiological wear and tear resulting from chronic stress, was another biological mechanism identified in the reviewed studies. Four studies explored this concept in the context of social adversity and cognitive outcomes. For instance, research by Fazeli et al. [39] demonstrated that allostatic load mediated the relationship between SES and neurocognitive function in older adults living with HIV, supporting the notion that prolonged exposure to social adversity may lead to physiological dysregulation, which in turn exacerbates cognitive decline [39]. Similarly, Krishnadas et al. [32] found that socioeconomic deprivation was associated with cortical structural changes, particularly in the Wernicke’s area, and that inflammatory markers mediated this relationship [32]. Conversely, Akrivos et al. [29] and De Looze et al. [37] reported that while social adversity was associated with poorer cognitive performance, allostatic load explained only partly explained the proportion of this variance, suggesting that other mechanisms may be more central to these associations [29, 37]. These findings point to the need for a more nuanced understanding of how allostatic load contributes to cognitive aging, particularly when considered alongside other factors such as inflammation and genetic predispositions, and importantly social and environmental exposures in early-life and across the life course [41, 42].

The role of genetics, epigenetics, and genetic aging markers in mediating the relationship between social adversity and cognitive outcomes emerged as another important area of interest. Several studies investigated telomere length (LTL), DNA methylation, and epigenetic aging as potential biological mediators. Huang et al. [31] found that shorter LTL mediated the relationship between low SES and cognitive decline, providing support for the hypothesis that social adversity accelerates biological aging. However, other studies, such as De Looze et al. [37], did not find LTL to mediate the association between perceived stress and cognitive outcomes, suggesting that epigenetic mechanisms may be context-dependent. Notably, studies examining DNA methylation, including those by Lynch et al. [38] and Liang et al. [16], found that specific epigenetic changes, such as those linked to loneliness and social isolation, were associated with poorer cognitive outcomes. For example, Lynch et al. [38] demonstrated that epigenetic aging, as measured by GrimAge, mediated the relationship between loneliness and cognitive decline. These findings suggest that epigenetic alterations could represent a critical biological pathway through which social adversity influences cognitive health, though more research is needed to establish causal links and to identify which epigenetic markers are most predictive of cognitive outcomes.

Overall, the differing findings from some reviewed studies likely reflect heterogeneity in study populations as well as the differences in types of social adversities assessed. For instance, some studies used objective indicators of SES, while others relied on subjective measures like perceived stress or loneliness, which may engage different biological pathways. Some studies also measured only one specific cognitive domain as opposed to overall cognition. These differences underscore the importance of further future studies with standardized measurement approaches to better capture the influence of biological mediators on this association between social adversity and cognitive aging.

Notably, while inflammation, allostatic load, and epigenetics represent promising biological pathways linking social adversity to cognitive decline, the reviewed studies also highlight the need for more comprehensive models that integrate multiple biological, psychological, and social factors. Many of the studies included in this review focused on a single biological mechanism or social factor, often in isolation, and did not consider the potential for complex interactions between these variables. For instance, while inflammation was a common mediator in the studies examining social isolation, its effect was not always consistent across different cognitive domains or social contexts [30, 35]. Similarly, while allostatic load and SES were found to be associated with cognitive outcomes, the magnitude of the relationship was often modest and varied depending on the individual’s specific life circumstances [29]. This suggests that future research should adopt a more integrative, multidimensional approach that considers the dynamic interplay between genetic, physiological, and social factors in shaping cognitive health [43]. Longitudinal studies with larger and more diverse populations are particularly needed to establish causal relationships and to explore how these pathways evolve over time, especially in light of the aging process. By advancing our understanding of the complex mechanisms through which social adversity affects cognitive aging, researchers can help inform interventions aimed at mitigating the impact of social inequalities on cognitive health in older adults.

As well, this study did not explore if similar biological mediators exist for the well-known association between early-life adversity, such as low SES, childhood trauma, chronic stress, and effects on cognitive function in later adulthood [44, 45]. Adverse childhood experiences (ACEs), such as poverty, neglect, or family instability, have been associated with lasting alterations in stress regulation, inflammation, and brain development, which may predispose individuals to accelerated cognitive aging. For instance, studies have shown that early-life socioeconomic disadvantage is linked to altered HPA axis functioning and pro-inflammatory phenotypes, which have been implicated in later-life cognitive impairment [46, 47]. For future studies, it would be important to apply a life course perspective and consider how accumulation of adversity influences cognitive aging trajectories. Additionally, the role of intersectional factors such as gender and race was only mentioned in one study by Fazeli et al. Incorporating life course epidemiology and intersectionality into future research is essential for identifying sensitive periods for intervention and better understanding disparities in cognitive aging outcomes. As well, future studies should stratify analyses or test for effect modification to identify populations most vulnerable to biologically mediated effects of social adversity on cognition, taking into consideration factors such as sex, ethnicity, and ACEs.

There are several strengths to our study. Our search strategy was developed in collaboration with a trained research librarian to identify the optimal data sources and search terms for a comprehensive review. Quality assessment showed that all the studies included in our review achieved over seven points on the nine-point NOS scale. This is largely since most studies employed pre-existing, large-scale, nationally representative data, minimizing selection bias in participants and confirmation bias in data analysis. Our study also has some limitations including the modest number of studies that were eligible for inclusion; we had also excluded studies in languages other than English. Also, the heterogeneity in the studies included in terms of social adversity, cognitive domains, and biological factors may have limited the ability to compare the included studies. The included studies are predominantly cross-sectional studies, which limits the ability to infer causal or temporal relationships, and future research that focuses on longitudinal cohort designs would be meaningful for establishing causality. Lastly, given that our scoping review has chosen to focus on the aforementioned biological mediators of inflammation, allostatic load, and genetics factors, we have not explored other plausible pathways, such as neurotrophic factors including brain-derived neurotrophic factor, oxidative stress, and microbiome-gut-brain axis, which should be investigated in future reviews [48–50].

### Clinical and Health Policy Implications

The findings of this scoping review have some clinical and health policy implications, particularly for addressing cognitive decline and dementia in aging populations. Clinically, the recognition of social adversity—such as low SES, social isolation, and chronic stress—as significant risk factors for cognitive decline highlights the need for healthcare providers to take a holistic, biopsychosocial approach when assessing and managing older adults. Incorporating social assessments into routine cognitive screenings could help identify at-risk individuals early, allowing for timely interventions such as community programs to promote social engagement aimed at mitigating the effects of social adversity on brain health [51]. Additionally, given the role of biological mediators such as inflammation and allostatic load, clinicians should consider implementing strategies to reduce inflammation and managing stress in older adults, including lifestyle interventions like physical activity, social engagement, and stress management techniques, which have been shown to improve both physical and cognitive outcomes [5, 52]. From a policy perspective, these findings emphasize the need for public health initiatives that address the social determinants of health, particularly in vulnerable aging populations. Policies that promote social connectedness, economic stability, and access to mental health resources could help reduce the burden of cognitive decline and dementia, particularly in communities facing higher levels of socioeconomic deprivation. Moreover, policies aimed at reducing health disparities by providing equitable access to healthcare and social services for older adults such as culturally tailored community programs are crucial in mitigating the cognitive impacts of social adversity [53, 54]. The integration of social interventions, such as community-based programs that foster social networks and reduce isolation, could complement traditional medical approaches to aging and cognitive health. In summary, a multidisciplinary approach—incorporating both social and biomedical interventions—should be prioritized in clinical practice and health policy to optimize cognitive aging and improve the quality of life for older adults.

### Conclusion

In conclusion, this scoping review underscores the complex and multifactorial nature of the relationship between social adversity and cognitive health in aging, highlighting the role of biological mediators such as inflammation, allostatic load, and epigenetic changes. While substantial evidence supports the influence of social factors—such as SES, loneliness, and psychosocial stress—on cognitive decline, the specific biological pathways that mediate these associations remain only partially understood. Our review reveals both the promise and the limitations of current research, pointing to the need for more comprehensive, longitudinal studies that integrate biological, psychological, and social perspectives to fully capture the dynamic interactions between these factors. By addressing the existing knowledge gaps, future research can better inform interventions and public health strategies aimed at promoting cognitive resilience and reducing the burden of cognitive decline among aging populations, particularly in socially disadvantaged groups. Ultimately, a deeper understanding of these pathways will be critical for developing targeted interventions that foster healthy aging and improve cognitive outcomes for individuals across the life course.
