# Supplementary material for: Biological Mechanisms Linking Social Adversity and Cognition
Source: Public Health Rev. 2025 Nov 5;46:1608740. doi: 10.3389/phrs.2025.1608740 (PMC12636236; doi:10.3389/phrs.2025.1608740)
Supplement: Supplementary file 1 [file Supplementaryfile1.docx]

**S1 Appendix. Complete Search Strategy**

| Term 1 social adversity | Term 2 cognitive health | Term 3 Biological Mechanisms (allostatic load, inflammation, or genetics) |
| --- | --- | --- |
| social capital OR structural social capital OR  cognitive social capital OR  social support OR  social participation  OR social cohesion  OR  Social network OR  Social trust OR  Reciprocity OR  Sense of belonging OR  Loneliness OR  Social engagement OR  Social integration OR  Social relationship OR  Bonding social capital OR  Bridging social capital OR  Linking social capital OR  Psychosocial stress OR  Allostatic load OR  Socioeconomic status OR  Social adversity | Neurocognitive health OR  cognitive health OR  cognition OR  cognitive impairments OR  executive functions OR  memory OR  psychomotor speed OR  verbal fluency OR  working memory OR  flexible thinking OR  inhibitory control OR  shifting OR  mental flexibility OR  attention | Inflammaging OR  inflammation OR  Inflammasome OR chronic inflammation OR  Epigenetic OR  Telomere OR  Telomere length OR  epigenetic clock OR  Allostatic load  Cortisol OR |

1722 articles imported, 590 duplicates removed.

**Databases**

- Medline (from Ovid) - 616
- Embase (from Ovid) - 1080
- psychINFO (from Ovid) - 248
- SCOPUS – 368

References of review articles and studies were also manually searched for additional studies.

**Cutoff date: March 5, 2024**

| **Level 1 screening (title and abstract)** |
| --- |
| - **Age: over 18** - **Country: any** - **Language: English/translated** - **Must contain a measure of cognition or a measure of inflammaging** - **Must contain a measure of social adversity** |

| **Inclusion criteria** | **Exclusion criteria** |
| --- | --- |
| - **Age: over 18** - **Country: any** - **Language: English** - **Study type: any primary research** - **Must contain a measure of cognition or a measure of biological mechanism** - **Must contain a measure of social adversity (low social capital, low SES, psychosocial stress)** | - **Reviews/meta-analysis** - **Studies focused on pediatric population or early childhood events/exposures** - **Studies in languages other than English** - **Mentions of stress that does not pertain to psychosocial environment (eg. Acute, lab-induced stress or psychiatric/mood disorders)** - **Non-human study** |

**Medline**


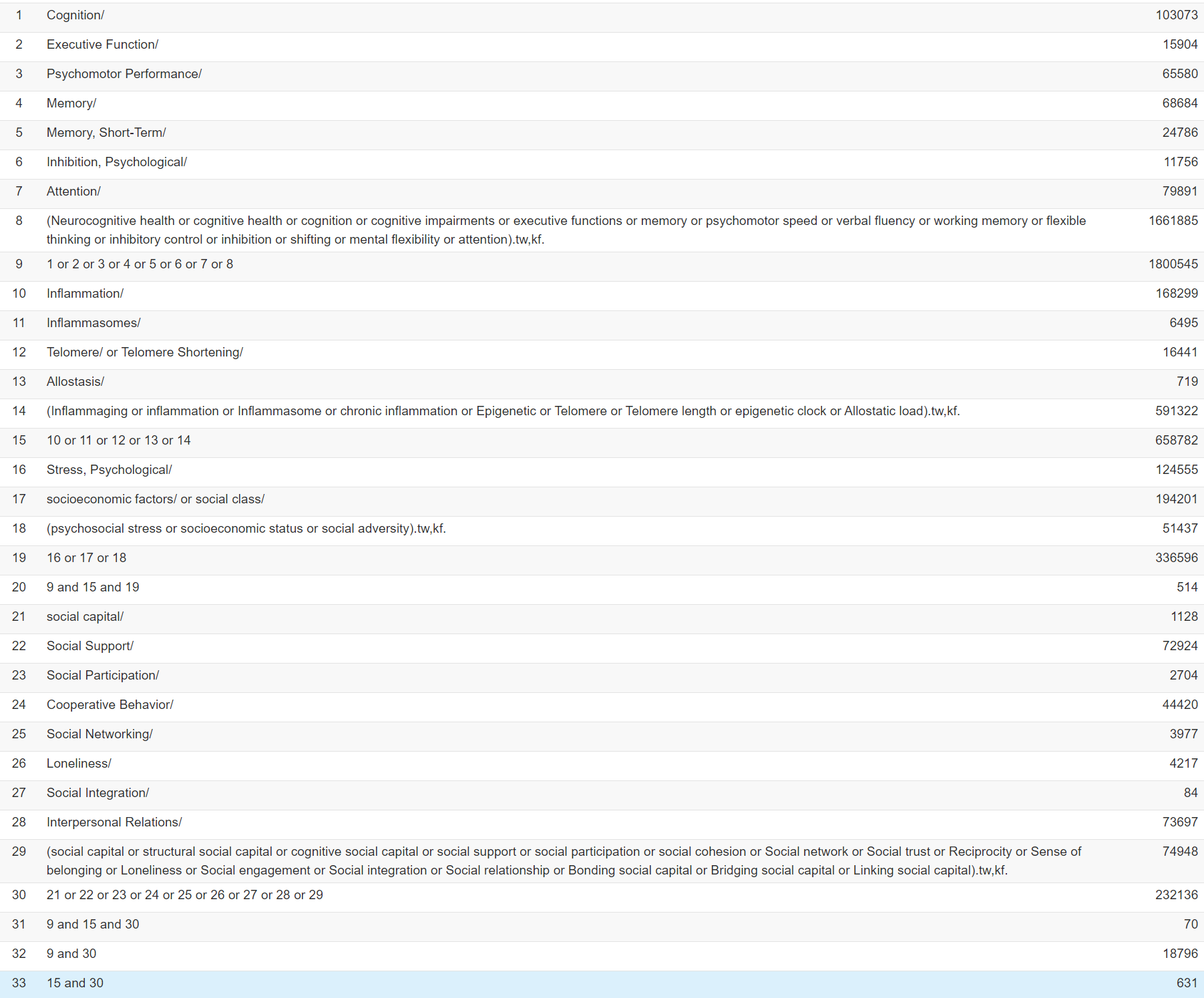


**EMBASE**


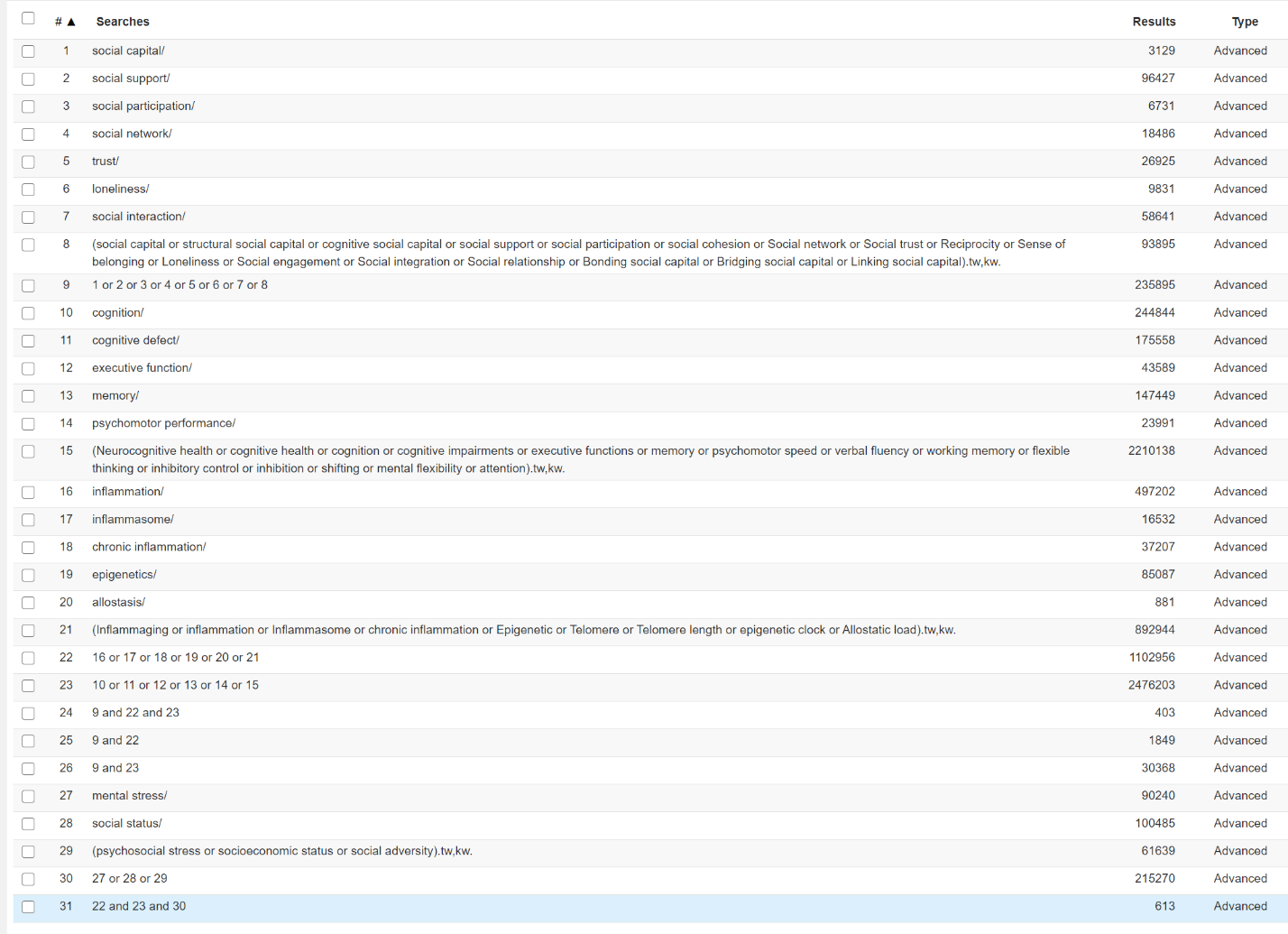


**PsychINFO**


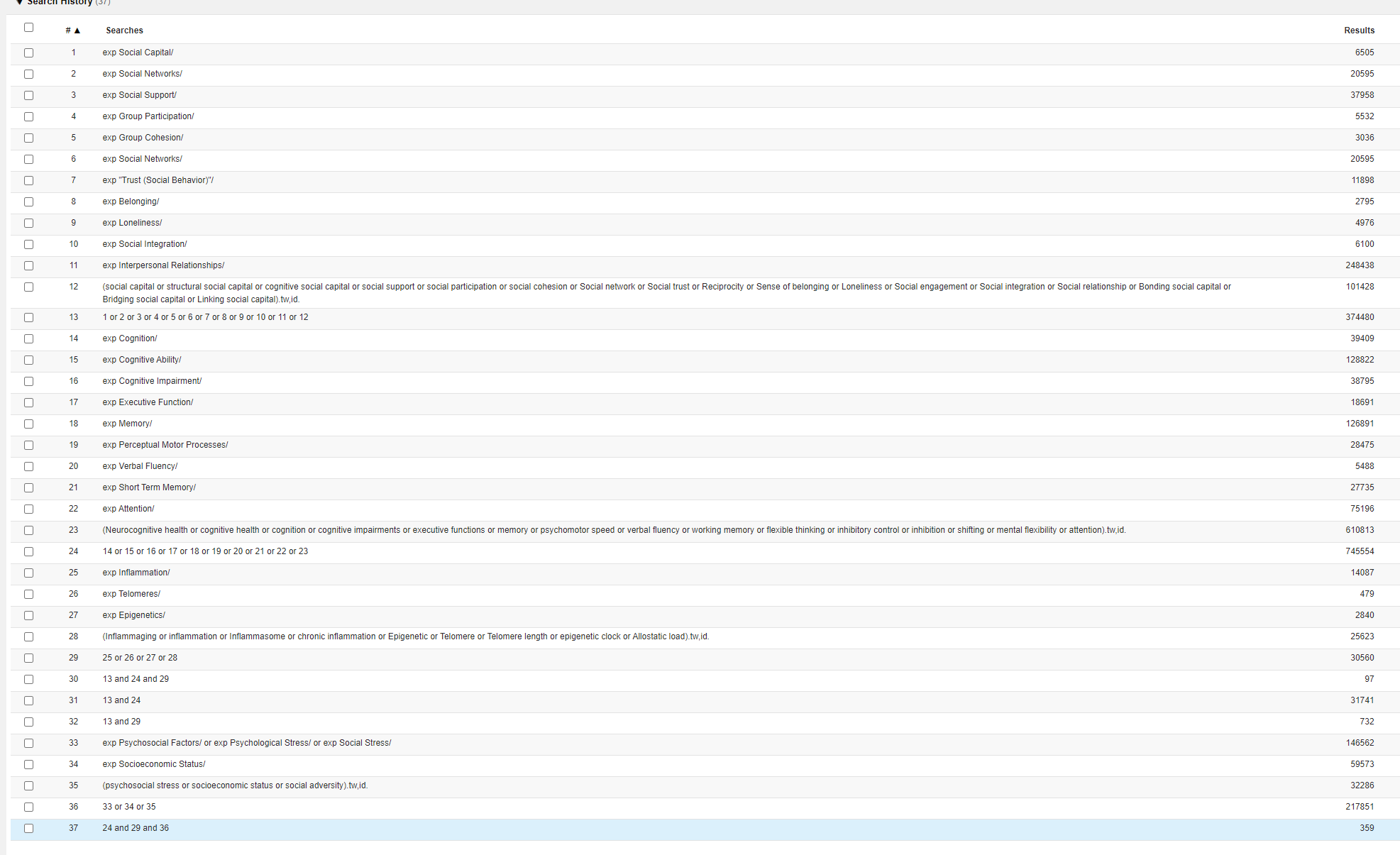


**Scopus**

( TITLE-ABS-KEY ( "social capital" OR "structural social capital" OR "cognitive social capital" OR "social support" OR "social participation" OR "social cohesion" OR "Social network" OR "Social trust" OR "Reciprocity" OR "Sense of belonging" OR "Loneliness" OR "Social engagement" OR "Social integration" OR "Social relationship" OR "Bonding social capital" OR "Bridging social capital" OR "linking social capital" ) AND TITLE-ABS-KEY ( "Neurocognitive health" OR "cognitive health" OR "cognition" OR "cognitive impairment*" OR "executive function*" OR "memory" OR "psychomotor speed" OR "verbal fluency" OR "working memory" OR "flexible thinking" OR "inhibitory control" OR "inhibition" OR "shifting" OR "mental flexibility" OR "attention" ) )

( TITLE-ABS-KEY ( "social capital" OR "structural social capital" OR "cognitive social capital" OR "social support" OR "social participation" OR "social cohesion" OR "Social network" OR "Social trust" OR "Reciprocity" OR "Sense of belonging" OR "Loneliness" OR "Social engagement" OR "Social integration" OR "Social relationship" OR "Bonding social capital" OR "Bridging social capital" OR "linking social capital" ) AND TITLE-ABS-KEY ( "Neurocognitive health" OR "cognitive health" OR "cognition" OR "cognitive impairment*" OR "executive function*" OR "memory" OR "psychomotor speed" OR "verbal fluency" OR "working memory" OR "flexible thinking" OR "inhibitory control" OR "inhibition" OR "shifting" OR "mental flexibility" OR "attention" ) AND TITLE-ABS-KEY ( "Inflammaging" OR "inflammation" OR "Inflammasome" OR "chronic inflammation" OR "Epigenetic*" OR "Telomere*" OR "Telomere length" OR "epigenetic clock" OR "Allostatic load" ) )

( TITLE-ABS-KEY ( "Inflammaging" OR "inflammation" OR "Inflammasome" OR "chronic inflammation" OR "Epigenetic*" OR "Telomere*" OR "Telomere length" OR "epigenetic clock" OR "Allostatic load" ) AND TITLE-ABS-KEY ( "Neurocognitive health" OR "cognitive health" OR "cognition" OR "cognitive impairment*" OR "executive function*" OR "memory" OR "psychomotor speed" OR "verbal fluency" OR "working memory" OR "flexible thinking" OR "inhibitory control" OR "inhibition" OR "shifting" OR "mental flexibility" OR "attention" ) AND TITLE-ABS-KEY ( "psychosocial stress" OR "socioeconomic status" OR "social adversit*" ) )

**S2 Appendix. Results of Newcastle-Ottawa Quality Assessment Scale**

|  | Selection |  |  |  | Comparability |  | Outcome |  |  |
| --- | --- | --- | --- | --- | --- | --- | --- | --- | --- |
|  | Representativeness of the exposed cohort | Selection of the non exposed cohort | Ascertainment of exposure | Demonstration that outcome of interest was not present at start of study | Study controls for age and sex | Study controls for additional factors | Assessment of outcome | Was follow-up long enough for outcomes to occur | Adequacy of follow up of cohorts |
| Akrivos 2020 | * | * | * | * | * | * | * | * | * |
| Boss 2016 | * | * | * | 0 | * | * | * | * | * |
| Fazeli 2020 | * | * | 0 | * | * | * | * | * | * |
| Foverskov 2020 | * | * | * | * | * | * | * | * | * |
| Hatton 2018 | * | * | * | 0 | * | * | * | * | * |
| Huang 2019 | * | * | 0 | * | * | 0 | * | * | * |
| Krishnadas 2013 | * | * | * | 0 | * | 0 | * | * | * |
| Wilson 2007 | * | * | * | * | * | * | * | * | * |
| Molesworth 2015 | * | 0 | 0 | * | * | * | * | * | * |
| Phillips 2020 | * | 0 | * | * | * | * | * | * | * |
| Lynch 2023 | * | * | * | * | * | * | * | * | * |
| Liang 2023 | * | * | * | * | * | * | * | * | * |
| De Looze 2024 | * | * | * | * | * | * | * | * | * |
| Qi 2023 | * | * | * | * | * | * | * | * | * |
| Malatyali 2023 | * | * | * | * | * | * | * | * | * |
